# Rare case of severe non-calcific aortic stenosis in an achondroplastic dwarf: surgical consideration

**DOI:** 10.1093/icvts/ivab335

**Published:** 2021-11-29

**Authors:** Tsubasa Mikami, Satoshi Kainuma, Koichi Toda, Yoshiki Sawa

**Affiliations:** Department of Cardiovascular Surgery, Osaka University Graduate School of Medicine, Suita, Osaka, Japan

**Keywords:** Achondroplasia, Aortic stenosis, Aortic valve replacement, Aortic root enlargement

## Abstract

A 41-year-old patient with achondroplasia who had undergone surgery for congenital aortic stenosis >20 years ago presented with dyspnoea due to severe aortic stenosis. Computed tomography confirmed a small (16-mm) aortic annulus, thickened aortic valve leaflets without calcification and subaortic fibrous tissues. Intraoperatively, 3 non-calcific aortic leaflets were observed. Enlargement of the aortic root using a modified Manouguian technique for good exposure enabled the resection of subaortic tissues and replacement of the valve with a mechanical valve. The aortic root can be extremely small in patients with aortic stenosis and achondroplasia. The anatomy of the aortic root should be carefully assessed to enable appropriate surgical planning.

## INTRODUCTION

Achondroplasia, the most common form of human dwarfism, involves an increased risk of cardiovascular diseases [1]. Here, we report a rare case of achondroplasia with severe non-calcific aortic stenosis (AS) treated using aortic valve replacement combined with aortic root enlargement. We also review the literature for cases of achondroplasia with severe AS and focus on histological findings of the resected aortic valve.

## CASE REPORT

This study was approved by the Institutional Review Board of Osaka University Hospital (Approval No. 16105). The patients’ legal guardians provided written informed consent to use his medical records.

**Figure 1:**
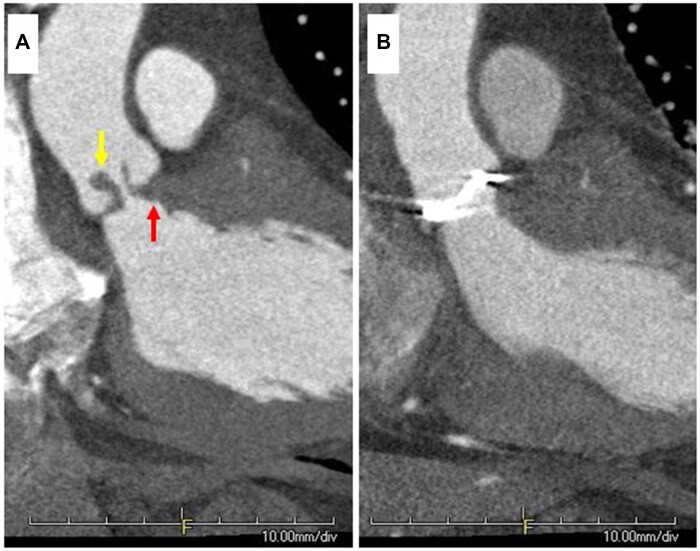
Preoperative (**A**) coronal and (**B**) axial computed tomography scans showing a small aortic annulus, thickened aortic valve leaflets without calcification (yellow arrow) and subaortic fibrous tissue (red arrow). Extracted aortic valves showing no calcification on (**C**) macroscopic and (**D**) microscopic pathological examinations. (**E**) Postoperative computed tomography scan showing no left ventricular outflow tract stenosis.

A 41-year-old man with achondroplasia (height, 128.9 cm; weight, 55.7 kg; body surface area, 1.34 m^2^) who had undergone aortic subvalvular tissue resection and aortic valve commissurotomy for congenital AS at the age of 14 years was admitted with dyspnoea. Echocardiography showed an ejection fraction of 70% and severe AS (maximum transvalvular gradient, 68 mmHg; peak velocity, 4.0 m/s; aortic valve area, 0.68 cm^2^) without any other congenital heart disease. Computed tomography revealed a small aortic annulus (diameter, 16 mm), thickened non-calcific aortic valve leaflets, and subaortic fibrous tissue (Fig. 1A). The patient was scheduled to undergo repeat surgery.

**Figure 2:**
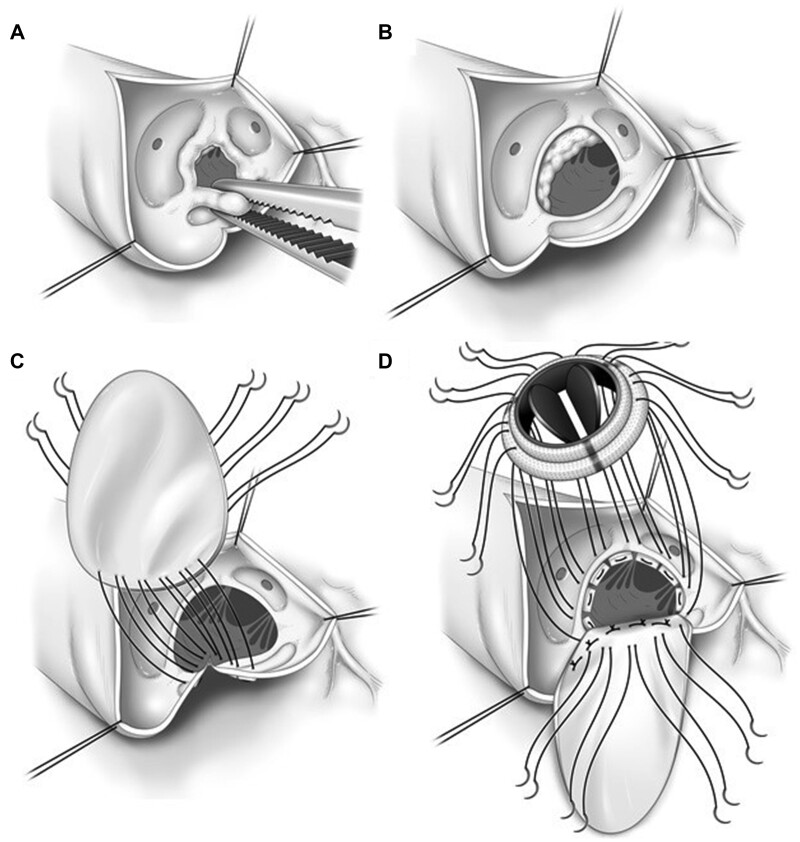
Schemas of the operative findings. (**A**) Thickened aortic valve leaflets without calcification, (**B**) subaortic fibromuscular tissues partially overhanging the left ventricular outflow tract, (**C**) aortic root enlargement with use of a bovine pericardium (Edwards Lifesciences, Irvine, CA, USA) and (**D**) aortic valve replacement using a mechanical valve.

During surgery, after cardiac arrest was achieved, we performed an oblique aortotomy and extracted the aortic valve, which was tricuspid, fibrous and markedly thickened without calcification (Fig. 2A and Supplementary Material, Fig. S1). The subaortic fibromuscular tissues, which were continuous, located beneath the aortic annulus, and partially overhanging the left ventricular outflow tract, were then meticulously resected. As the aortic annulus diameter was only 15 mm (Fig. 2B), the aortotomy was extended across the aortic annulus through the non-coronary Valsalva sinus and over the aortomitral continuity and the aortic annulus diameter was enlarged to 17 mm (Fig. 2C). A tear-shaped bovine pericardial patch was used to reconstruct the aortic annulus and augment aortic root. Then, the aortic valve was replaced with a 16-mm ATS-AP mechanical valve (ATS Medical Inc., Minneapolis, MN, USA) (Fig. 2C and 2D). Postoperative echocardiography showed good mechanical valve function (trans-prosthesis maximum gradient, 14 mmHg). The patient was uneventfully discharged and remained well for the subsequent 1 year.

## DISCUSSION

Although achondroplasia itself is not a contraindication for cardiac surgery [1], only 3 cases of aortic valve replacement for AS in patients with achondroplasia have been reported to date (see Supplementary Material, Table S1). Previous reports and our case illustrate that an extremely small aortic root complicates the treatment of AS with achondroplasia (Supplementary Material, Table S1). In our case, a modified Manouguian technique helped aggressive resection of the subaortic tissue and enlargement of the annulus, allowing left ventricular outflow tract stenosis elimination and mechanical valve implantation (Fig. 1B). These findings support the need for aortic root enlargement in patients with AS and achondroplasia.

Our technique for aortic root enlargement is a slight modification of the original Manouguian technique and might have had 2 potential benefits in the current case. First, as our patient had a significantly small aortic root, the suturing of a tear drop-shaped bovine pericardial patch to the aortic wall at the side of the left coronary sinus of Valsalva using the original Manouguian technique would have interfered with the orifice of the left coronary artery. The modified procedure enabled us to maintain enough space between the orifice of left coronary artery and the patch sutures and safely enlarge the aortic root. Second, the length of the incision from the aortic annulus to the mitral valve could be shortened. Therefore, our method minimized the area of subaortic tissue and mitral annulus to be reconstructed and prevented the incision from affecting the left atrium while enabling sufficient enlargement of the aortic root.

The pathological findings of extracted aortic valves in achondroplasia vary among reports. Scafuri *et al.* [2] and Baikoussis *et al.* [3] observed heavy calcification of the aortic valves, whereas Huang *et al.* [4] found fibrous and markedly thickened non-calcific aortic valves similar to our findings. This is a notable discrepancy because non-calcific AS might not be indicated for transcatheter aortic valve implantation as in the present case. The only common factor between the case reported by Huang *et al.* [4] and our case is that surgery was performed at a relatively young age compared to the other patients (Supplementary Material, Table S1). Accumulating experience and knowledge are necessary to increasing our understanding of the potential association of achondroplasia with fibrous and thickened but non-calcific aortic valves.

## CONCLUSIONS

The aortic root can be extremely small in patients with AS and achondroplasia who undergo aortic valve replacement; thus, careful assessment of the aortic root anatomy is crucial. Aortic root enlargement is a useful strategy that enables the implantation of a larger valve than what the native annulus would normally accept.

## SUPPLEMENTARY MATERIAL

Supplementary material is available at *ICVTS* online.

**Conflict of interest:** none declared. 

##  

### Reviewer Information

Interactive CardioVascular and Thoracic Surgery thanks Nikolaos G. Baikoussis, Jae Hwan Choi, Antonios Kallikourdis, Gianluca Lucchese and the other, anonymous reviewer(s) for their contribution to the peer review process of this article.

## Supplementary Material

ivab335_Supplementary_Data
