# Supplementary material for: Rare case of severe non-calcific aortic stenosis in an achondroplastic dwarf: surgical consideration
Source: Interact Cardiovasc Thorac Surg. 2021 Nov 29;34(3):495–7. doi: 10.1093/icvts/ivab335 (PMC8860432; doi:10.1093/icvts/ivab335)
Supplement: ivab335_Supplementary_Data [file ivab335_supplementary_data.zip › Supplementary Data Table S1.docx]

Supplementary Table S1. Surgical cases of aortic stenosis in patients with achondroplasia

| Authors | Age  (years) | Sex | Surgical  procedures | Valve prosthesis | Aortic annulus  (mm) | Aortic valve  findings | Follow-up  duration (months) |
| --- | --- | --- | --- | --- | --- | --- | --- |
| Scafuri A,  *et al*. [2] | 56 | Female | AVR,  aortic root enlargement | 16-mm  CarboMedics | 10 | Calcified | 8 |
| Baikoussis NG,  *et al*. [3] | 58 | Female | AVR,  aortic root dilation | Perceval S | 11 | Calcified | N/A |
| Huang WY,  *et al*. [4] | 39 | Female | Aortic root replacement | Allograft | 14 | Fibrous,  markedly thickened | N/A |
| Mikami T,  *et al*.  [present case] | 41 | Male | AVR,  aortic root enlargement | 16-mm  ATS-AP | 15 | Fibrous,  markedly thickened | 12 |

AVR, aortic valve replacement; N/A, not applicable
