# Supplementary figures and images for: Rare case of severe non-calcific aortic stenosis in an achondroplastic dwarf: surgical consideration
Source: Interact Cardiovasc Thorac Surg. 2021 Nov 29;34(3):495–7. doi: 10.1093/icvts/ivab335 (PMC8860432; doi:10.1093/icvts/ivab335)

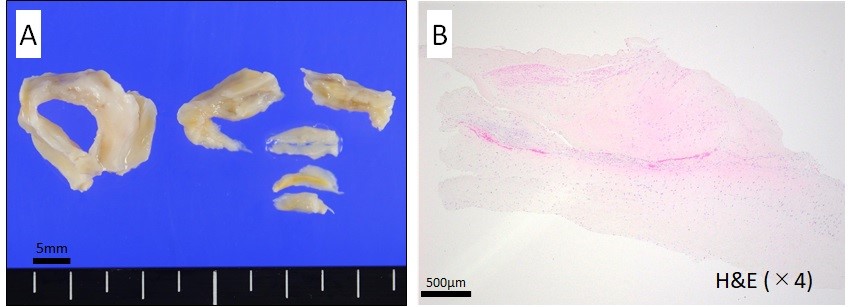

Supplement: ivab335_Supplementary_Data [file ivab335_supplementary_data.zip › Figure S1.jpg]
