# Supplementary material for: Rare case of severe non-calcific aortic stenosis in an achondroplastic dwarf: surgical consideration
Source: Interact Cardiovasc Thorac Surg. 2021 Nov 29;34(3):495–7. doi: 10.1093/icvts/ivab335 (PMC8860432; doi:10.1093/icvts/ivab335)
Supplement: ivab335_Supplementary_Data [file ivab335_supplementary_data.zip › Supplementary Data Figure S1.doc]

**Figure legend**

Supplementary Figure S1. Extracted aortic valves showing no calcification on (**A**) macroscopic and (**B**) microscopic pathological examinations.
